# Supplementary material for: Maternal smoking behaviour across the first two pregnancies and small for gestational age birth: Analysis of the SLOPE (Studying Lifecourse Obesity PrEdictors) population-based cohort in the South of England
Source: PLoS One. 2021 Nov 18;16(11):e0260134. doi: 10.1371/journal.pone.0260134 (PMC8601508; doi:10.1371/journal.pone.0260134)
Supplement: S1 Table — (DOCX) [file pone.0260134.s001.docx]

**S1 Table: Sensitivity analysis showing the effect of using different minimal adjustment sets on the adjusted odds ratios calculated in Model 1 in the full sample**

| **Model^¶^** | **Heavier smoker** | **Smoker** | **Smoker increased** | **Smoker reduced** | **Smoker P2^1^** | **Smoker P1^2^** | **Other smoker^3^** | **Ex-smoker** |
| --- | --- | --- | --- | --- | --- | --- | --- | --- |
| Adjusted odds ratios (aORs) | | | | | | | | |
| 1^†^ | 3.55 | 2.44 | 2.70 | 2.44 | 2.11 | 1.50 | 1.11 | 0.89 |
| Differences to the aORs for Model 1 in each of the models run with different adjustment sets^¶^ | | | | | | | | |
| A^†^ | 0.04 | -0.03 | 0.02 | 0.06 | 0.01 | 0.03 | 0.01 | 0.00 |
| B^†^ | -0.01 | -0.03 | 0.01 | 0.04 | 0.01 | 0.03 | 0.01 | 0.00 |
| C^†^ | 0.00 | -0.03 | 0.03 | 0.05 | 0.01 | 0.03 | 0.01 | 0.01 |
| D^‡^ | 0.13 | 0.04 | 0.19 | 0.06 | 0.07 | 0.00 | -0.01 | 0.00 |
| E^‡^ | 0.02 | -0.01 | 0.11 | 0.05 | 0.05 | -0.01 | -0.01 | 0.00 |
| F^‡^ | 0.15 | 0.05 | 0.21 | 0.07 | 0.08 | 0.00 | 0.00 | 0.00 |
| G^‡^ | 0.14 | 0.04 | 0.20 | 0.07 | 0.08 | 0.00 | 0.00 | 0.00 |
| H^‡^ | 0.04 | 0.00 | 0.14 | 0.06 | 0.05 | 0.00 | -0.01 | 0.00 |
| I^‡^ | 0.04 | 0.00 | 0.13 | 0.06 | 0.05 | -0.01 | -0.01 | 0.00 |
| J^§^ | 0.16 | 0.03 | 0.22 | 0.11 | 0.10 | 0.03 | 0.01 | 0.01 |
| K | 0.05 | -0.01 | 0.15 | 0.09 | 0.07 | 0.02 | 0.01 | 0.01 |
| L^§^ | 0.18 | 0.04 | 0.25 | 0.12 | 0.10 | 0.03 | 0.01 | 0.01 |
| M^§^ | 0.17 | 0.03 | 0.24 | 0.12 | 0.10 | 0.03 | 0.01 | 0.01 |
| N | 0.08 | 0.00 | 0.18 | 0.11 | 0.08 | 0.02 | 0.01 | 0.01 |
| O | 0.07 | -0.01 | 0.18 | 0.11 | 0.08 | 0.02 | 0.01 | 0.01 |
| P^§^ | 0.13 | 0.03 | 0.23 | 0.10 | 0.09 | 0.03 | 0.01 | 0.01 |
| Q | 0.02 | -0.01 | 0.16 | 0.08 | 0.07 | 0.02 | 0.00 | 0.01 |
| R^§^ | 0.15 | 0.04 | 0.25 | 0.11 | 0.10 | 0.03 | 0.01 | 0.01 |
| S^§^ | 0.14 | 0.03 | 0.25 | 0.11 | 0.10 | 0.03 | 0.01 | 0.01 |
| T | 0.04 | 0.00 | 0.19 | 0.11 | 0.07 | 0.02 | 0.01 | 0.01 |
| U | 0.04 | -0.01 | 0.18 | 0.10 | 0.07 | 0.02 | 0.01 | 0.01 |
| 1. A smoker at the first ANA for P2 who was not smoking at the first ANA for P1  2. A smoker at the first ANA for P1 who stopped before the first ANA for P2 3. A smoker later in P1 or between pregnancies; not smoking at the first ANA for P1 or P2  † These models had missing data for variables in 76 records  ‡ These models had missing data for variables in 5 records  § These models had missing data for variables in 1 record  ¶ All models adjusted for maternal ethnicity, length of the IPI, highest level of maternal educational attainment and previous SGA birth. The models additionally adjusted for:  **Model 1** Maternal employment, P1 BMI, P1 GDM, P1 age, P1 folic acid supplementation, P1 gestational hypertension, P1 infertility treatment, P1 partnership status  **Model A** Maternal employment, P1 BMI, P1 GDM, P1 age, P1 folic acid supplementation, P1 gestational hypertension, P1 partnership status, P2 BMI, P2 infertility treatment, P2 partnership status  **Model B** Maternal employment, P1 BMI, P1 age, P1 folic acid supplementation, P1 gestational hypertension, P1 infertility treatment, P1 partnership status, P2 BMI, P2 GDM  **Model C** Maternal employment, P1 BMI, P1 age, P1 folic acid supplementation, P1 gestational hypertension, P1 partnership status, P2 BMI, P2 GDM, P2 infertility treatment, P2 partnership status  **Model D** P1 BMI, P1 GDM, P1 age, P1 folic acid supplementation, P1 gestational hypertension, P1 infertility treatment, P1 partnership status, P2 age  **Model E** P1 BMI, P1 GDM, P1 age, P1 gestational hypertension, P1 infertility treatment, P1 partnership status, P2 age, P2 folic acid supplementation  **Model F** P1 BMI, P1 GDM, P1 folic acid supplementation, P1 gestational hypertension, P1 infertility treatment, P1 partnership status, P2 age, P2 partnership status  **Model G** P1 BMI, P1 GDM, P1 folic acid supplementation, P1 gestational hypertension, P1 infertility treatment, P2 age, P2 infertility treatment, P2 partnership status  **Model H** P1 BMI, P1 GDM, P1 gestational hypertension, P1 infertility treatment, P1 partnership status, P2 age, P2 folic acid supplementation, P2 partnership status  **Model I** P1 BMI, P1 GDM, P1 gestational hypertension, P1 infertility treatment, P2 age, P2 folic acid supplementation, P2 infertility treatment, P2 partnership status  **Model J** P1 GDM, P1 age, P1 folic acid supplementation, P1 gestational hypertension, P1 infertility treatment, P1 partnership status, P2 BMI, P2 age  **Model K** P1 GDM, P1 age, P1 gestational hypertension, P1 infertility treatment, P1 partnership status, P2 BMI, P2 age, P2 folic acid supplementation  **Model L** P1 GDM, P1 folic acid supplementation, P1 gestational hypertension, P1 infertility treatment, P1 partnership status, P2 BMI, P2 age, P2 partnership status  **Model M** P1 GDM, P1 folic acid supplementation, P1 gestational hypertension, P2 BMI, P2 age, P2 infertility treatment, P2 partnership status  **Model N** P1 GDM, P1 gestational hypertension, P1 infertility treatment, P1 partnership status, P2 BMI, P2 age, P2 folic acid supplementation, P2 partnership status  **Model O** P1 GDM, P1 gestational hypertension, P2 BMI, P2 age, P2 folic acid supplementation, P2 infertility treatment, P2 partnership status  **Model P** P1 age, P1 folic acid supplementation, P1 gestational hypertension, P1 infertility treatment, P1 partnership status, P2 BMI, P2 GDM, P2 age  **Model Q** P1 age, P1 gestational hypertension, P1 infertility treatment, P1 partnership status, P2 BMI, P2 GDM, P2 age, P2 folic acid supplementation  **Model R** 1 folic acid supplementation, P1 gestational hypertension, P1 infertility treatment, P1 partnership status, P2 BMI, P2 GDM, P2 age, P2 partnership status  **Model S** P1 folic acid supplementation, P1 gestational hypertension, P2 BMI, P2 GDM, P2 age, P2 infertility treatment, P2 partnership status  **Model T** P1 infertility treatment, P1 partnership status, P2 BMI, P2 GDM, P2 age, P2 folic acid supplementation, P2 partnership status  **Model U** P1 gestational hypertension, P2 BMI, P2 GDM, P2 age, P2 folic acid supplementation, P2 infertility treatment, P2 partnership status  **Abbreviations:** ANA, antenatal appointment; BMI, body mass index; P1, first pregnancy; P2, second pregnancy; SGA, small for gestational age (< 10^th^ percentile); OR, odds ratio; aOR, adjusted odds ratio; CI, confidence interval. | | | | | | | | |
