# Supplementary material for: Maternal smoking behaviour across the first two pregnancies and small for gestational age birth: Analysis of the SLOPE (Studying Lifecourse Obesity PrEdictors) population-based cohort in the South of England
Source: PLoS One. 2021 Nov 18;16(11):e0260134. doi: 10.1371/journal.pone.0260134 (PMC8601508; doi:10.1371/journal.pone.0260134)
Supplement: Attachment — Submitted filename: Response to reviewers 2021_09_14.docx [file pone.0260134.s002.docx]

**We thank the reviewers for their constructive comments; having responded to the points raised, the manuscript has been improved by their suggestions. We have included line numbers at each point based on the amended manuscript showing tracked changes. Please note that where tables have been revised, replacements have been included without tracked changes to aid clarity.**

**Comments to the Author**

Reviewer #1: Introduction:

This is an interesting retrospective study from a large prospectively collected dataset. In my opinion its value is as an exploratory study for hypothesis generation. Intuitively, I would have expected that women ceasing smoking between their first and second pregnancies would reduce their risk of SGA in the second pregnancy to around those of ex-smokers or never smokers. Instead, the authors have hypothesised that they have a continued increased risk for SGA when compared to never smokers without clarifying or justifying their hypothesis.

**Thank you, as mentioned in the Introduction, with this research we are particularly interested to examine whether the association between SGA birth and smoking extends beyond the period of the same pregnancy or whether exposure in a previous pregnancy or during the interconception period also carries risk of a SGA birth in a subsequent pregnancy. To better support our hypothesis and the justification for it, we have amended parts of the Introduction by removing two of the paragraphs and replacing them with the following [lines 66-72] :

“Changes to DNA methylation patterns have been seen in the placentas of women who quit smoking prior to pregnancy and a recent study suggests that tobacco exposure may cause long-term effects via the transmission of epigenetic marks to non-directly exposed placentas. A narrative review of epigenetic alterations due to maternal tobacco smoking in pregnancy concluded that there is increasing evidence to indicate that such alterations persist postnatally, but that there is also the suggestion of some reversibility of DNA methylation when stopping smoking either before or during pregnancy.”**

The fact they have shown an increased risk is hypothesis generating but I feel the conclusion is tentative and needs repeating in other data sets. There is significant potential for the result to be biased, based on patient recall of smoking status and the complicated stratification of smoking status that the authors have proposed.

**We agree that repeating this analysis in other datasets would strengthen the conclusions and have added the following to the Strengths and Weaknesses section [lines 420-421]:**

**“Repeating this analysis in other datasets will enable the comparison of results to see if our findings are replicated elsewhere”.**

**Potential biases which may have affected our ability to characterise the exposure correctly have already been discussed in this section [lines 410-416], and we have made some changes to the smoking categories as noted in response to the reviewer’s comments which follow to make the stratification clearer.**

I am also challenged by their decisions regarding covariates on which to adjust the odds ratios. The DAG diagram they present is overly complex and the software used to create a parsimonious set of adjusting covariates is not explained adequately. Their approach here negates any advantage of the DAG process to create a discussion and to explicitly explain and argue for the choices they have made. I would not feel comfortable with the current models without significant additional explanation of their decisions to adjust on covariates present at the start of the first pregnancy not the pregnancy of interest.

**Thank you, we agree that the DAG is complex but feel that this is necessary since we are considering variables which change across pregnancies. We have responded fully to the reviewer’s concerns about the DAG and the selection of covariates into the models in our response to the specific comments raised below.**

The hypothesis:

The stated hypothesis…. “that mothers who smoked in a previous pregnancy or who smoked between pregnancies have a higher risk of SGA in the second pregnancy compared to never smokers, even if they were not smoking during the second pregnancy”. Seems not to be an a priori hypothesis. It seems rather to have come out of the authors analysis of the data. The literature that the authors cite does not support this hypothesis.

I do agree with the authors stated aim… “to characterise maternal smoking behaviours across a mother’s first two pregnancies and examine the relation of smoking behaviours with second child’s risk of being born SGA”.

In my opinion the study would be better characterised as exploratory and hypothesis generating.
 **Thank you. As set out in our response to the first point made by the reviewer (above), we have provided specific justification in the Introduction section of the manuscript, and included additional references to support and clarify the rationale for our hypothesis. We hope that this will resolve these concerns.**

The 11 smoking categories for the Exposure are too complicated:

The following are clear and well defined:
• Heavy smoker
• Smoker
• Smoker increased
• Smoker reduced
• Smoker P2
• Ex smoker
• Never smoker
 **Thank you these categories have been retained throughout the revised manuscript.**

The following are likely to have significant overlap give that the classifications are self reported as well as being subject to recall bias.
• Smoker P1
• Serial quitter
• Smoker later in P1 or between pregnancies

I would like to see the impact of collapsing these into a single category of Smoked between pregnancies; quit prior to or on confirmation P2.

Exploratory analysis of collapsing these groups shows an unadjusted odds ratio for SGA when compared to the never smoked group of OR=1.08; 95% CI [.88-1.31].

**Thank you. We believe that there is a critical need for granularity of the classification used and that it is important to try to ascertain the effect of those who are actively smoking at the first antenatal appointment for the first pregnancy and compare it to those who report having stopped smoking at that appointment. Potential biases and under-reporting are discussed in the Discussion [lines 377-379] and Strengths and Limitations sections [lines 410-416].**

**When the three categories of Smoker P1, Serial quitter and Smoker later in P1 or between pregnancies are collapsed into one and compared to never smokers, the aOR is 1.25 [1.00, 1.56]. When Smoker P1 is retained and the other two categories are collapsed into one and compared to never smokers, the aOR for Smoker P1 is 1.50 [1.10, 2.03] and for the collapsed category is 1.11 [0.84, 1.45].

Based on the reviewer’s comments we have collapsed the categories of Serial quitter and Smoker later in P1 or between pregnancies into a single category which we have named “Other smoker”. We have retained the category of Smoker P1, based on our comments about the granularity of classification above.**

**The manuscript and analyses have been amended throughout to take account of this change. We have also taken the opportunity to simplify the descriptions in the tables and used appropriate notes within the tables. We now have headings of “Smoker P2” (table note: a smoker at the first ANA for P2 who was not smoking at the first ANA for P1), “Smoker P1” (table note: smoker at the first ANA for P1 who stopped before the first ANA for P2) and “Other smoker” (table note: smoker later in P1 or between pregnancies; not smoking at the first ANA for P1 or P2).**

As the aim is to examine the relationship between smoking behaviours between pregnancies and the outcomes for P2 whether the following behaviours could also be collapsed to simply things. Smoker – Heavy smoker + Smoker; Not smoking – Ex smoker+ Never smoker. I appreciate that there is a dose-response gradient for the smokers but as the studies stated aim is to examine changes across pregnancies, the inclusion of 11 categories makes the paper complex and more challenging to follow and a more parsimonious approach may help readers.

**We believe that it is useful to look separately at women who increase or decrease their reported level of smoking between pregnancies, since smoking reduction may be possible for some women where quitting may not. However, for the reviewer’s information, collapsing the four categories of women smoking at the start of both pregnancies into one gives an aOR of 2.68 [2.19, 3.28], compared to never smokers. This information could be included within the manuscript if it is considered to be useful.**

The use of Directed Acyclic Graph (DAG) technique:

I appreciate that the DAG technique allows for discussion by making underlying relations explicit. However, Figure 2 is incredibly complex and difficult to follow. Further, the use of the software package “DAGitty” to create a parsimonious set of factors to adjust for is poorly explained and has the feeling of a “black box” approach that is opaque to the reader.

At face value I struggle with the author’s or DAGitty’s choice to use variables (Age, Education, Infertility treatment, folic acid) from P1 for the adjusted odds ratio. I struggle even more with the use of complications in the first pregnancy (Gestational diabetes mellitus, Gestational hypertension).

I would appreciate a more explicit explanation of this decision and greater explanation of the DAGitty process and the intervening steps. A more parsimonious diagram of the final proposed causal model would help the reader decide whether they feel the approach and hence any results and conclusions drawn is appropriate.

**Thank you. We have taken the opportunity of revisiting the diagram for the DAG and have created the one shown at the end of this reply, which we believe is clearer. It includes all the variables that we consider to be confounders of the relationship between interpregnancy smoking change and SGA birth in the second child. References to maternal education and employment in the DAG are taken to be at the start of the first pregnancy.**

**Taking gestational hypertension as an example, you can see that we consider gestational hypertension in the first pregnancy to be a confounder since we hypothesise that it could influence smoking behaviour between pregnancies and before the second pregnancy (the main exposure). It potentially affects pregnancy 1 SGA birth, which in turn affects pregnancy 2 SGA birth (the main outcome). Pregnancy 1 hypertension also affects pregnancy 2 hypertension, which could be considered a mediator in this case.**

**We have run new sensitivity analysis where we used the other minimal adjustment sets identified by DAGitty to confirm that there is no difference in the effect estimates whichever minimal adjustment set is used. We do, however, completely take the reviewer’s point about the use of variables for complications arising in the first pregnancy (specifically gestational diabetes mellitus and gestational hypertension) and recognise that a number of second pregnancy variables could be mediators between interpregnancy smoking change and SGA birth in the second pregnancy (as per the gestational hypertension example above). We have therefore considered further the effect of such mediation through second pregnancy diagnoses of gestational diabetes and gestational hypertension and have now added a second layer of models to the analysis to take account of this. The pregnancy 2 variables adjusted for in the second set of models could be on the causal pathway, since for example, a change in interpregnancy smoking behaviour could influence gestational hypertension in the second pregnancy which then influences SGA birth in that pregnancy. In our analysis, Model 1 now adjusts for confounders and Model 2 for confounders and mediators.**

**The paragraphs discussing the DAG and the selection of the variables into the two sets of Models in the analysis now read as follows, and we hope that this now removes any opacity and allows the reader to better follow our approach [lines 162-198]:**

**“The association between change in smoking behaviour between pregnancies and the risk of SGA birth in the second pregnancy was examined by fitting logistic regression models predicting a binary outcome (SGA/not SGA). A minimal sufficient adjustment set of confounding variables was identified using a directed acyclic graph (DAG) constructed using DAGitty.net (Fig 2). The DAG illustrates the hypothesised confounding relationships by factors collected at the start of each pregnancy and explicitly identifies our assumptions using *a priori* causal knowledge. References to maternal education and employment in the DAG are taken to be those recorded at the start of the first pregnancy in our analysis.**

**A large number of minimal sufficient adjustment sets were identified using DAGitty.net. We selected a parsimonious set comprising maternal age, BMI, educational attainment, employment status, partnership status, folate supplementation and infertility treatment details collected at the start of the first pregnancy, diagnoses of gestational diabetes mellitus and gestational hypertension recorded during the first pregnancy, SGA birth in the first pregnancy, maternal ethnicity and the length of the interpregnancy interval (Model 1). The variables were complete in all but 72 cases. In 551 cases ethnicity was not recorded and has been included as “Not specified”.**

**Each minimal adjustment set identified should close all biasing paths, leaving only measured causal paths open. We used the other sets identified, some of which included covariates collected at the start of or during the second pregnancy, to confirm that there was no change to the results of our analyses and this sensitivity analysis is presented in Supplementary Table 1.**

**Whilst the minimal adjustment set used in this analysis consists mainly of covariates identified at the start of the first pregnancy, a number of second pregnancy covariates may be mediators of the effect of interpregnancy smoking change on SGA birth in the second pregnancy. Analysis was also therefore undertaken to take account of potential mediators. This analysis also followed a minimal adjustment set identified by DAGitty.net, but this time taking account of mediators. The identified adjustment set was the same as that identified for Model 1, but with gestational diabetes mellitus and gestational hypertension diagnosed in the second pregnancy in place of that for the first pregnancy and with the addition of maternal BMI recorded at the start of the second pregnancy (Model 2). The adjustment set for Model 2 should close all other measured causal paths with the exception of the effect of interpregnancy smoking change on SGA birth in the second pregnancy.”**

**Table 4 has been revised as follows to include results for Models 1 and 2 and the results section has now been updated as follows [lines 255-330]:**

**“Table 4 presents odds ratios for SGA birth in the second pregnancy according to the mother’s history of smoking and change in smoking behaviour between the first and second pregnancy, with Model 1 adjusting for confounders and Model 2 adjusting for confounders and mediators.**

#### **Table 4: The odds of small for gestational age birth (< 10^th^ percentile) in the second pregnancy**

|  | **Full sample** | | **Without previous SGA** | | **With previous SGA** | |
| --- | --- | --- | --- | --- | --- | --- |
|  | **n** | **Odds Ratios  (95% CI)** | **n** | **Odds Ratios  (95% CI)** | **n** | **Odds Ratios  (95% CI)** |
| **Heavier Smoker** | 330 |  | 256 |  | 74 |  |
| Unadjusted |  | **3.79 (2.84, 5.05)** |  | **3.66 (2.48, 5.41)** |  | **2.66 (1.64. 4.31)** |
| Model 1^†^ |  | **3.54 (2.55, 4.92)** |  | **3.53 (2.32, 5.38)** |  | **3.34 (1.96, 5.68)** |
| Model 2^‡^ |  | **3.57 (2.57, 4.97)** |  | **3.52 (2.31, 5.37)** |  | **3.54 (2.07, 6.08)** |
| **Smoker** | 786 |  | 623 |  | 163 |  |
| Unadjusted |  | **2.64 (2.12, 3.29)** |  | **2.48 (1.84, 3.36)** |  | **1.93 (1.35, 2.74)** |
| Model 1^†^ |  | **2.44 (1.89, 3.15)** |  | **2.43 (1.75, 3.39)** |  | **2.34 (1.56, 3.51)** |
| Model 2^‡^ |  | **2.43 (1.88, 3.14)** |  | **2.47 (1.77, 3.44)** |  | **2.23 (1.48, 3.35)** |
| **Smoker increased** | 344 |  | 267 |  | 77 |  |
| Unadjusted |  | **3.00 (2.21, 4.05)** |  | **2.88 (1.90, 4.37)** |  | **1.99 (1.22, 3.25)** |
| Model 1^†^ |  | **2.70 (1.92, 3.82)** |  | **2.84 (1.82, 4.44)** |  | **2.42 (1.41, 4.16)** |
| Model 2^‡^ |  | **2.75 (1.94, 3.88)** |  | **2.87 (1.84, 4.49)** |  | **2.51 (1.45, 4.32)** |
| **Smoker reduced** | 307 |  | 247 |  | 60 |  |
| Unadjusted |  | **2.63 (1.89, 3.67)** |  | **3.02 (1.97, 4.61)** |  | 1.50 (0.84, 2.65) |
| Model 1^†^ |  | **2.44 (1.68, 3.54)** |  | **2.98 (1.90, 4.67)** |  | 1.75 (0.94, 3.25) |
| Model 2^‡^ |  | **2.50 (1.72, 3.63)** |  | **3.05 (1.95, 4.78)** |  | 1.82 (0.97, 3.39) |
| **Smoker P2^1^** | 452 |  | 377 |  | 75 |  |
| Unadjusted |  | **2.09 (1.55, 2.82)** |  | **2.22 (1.50, 3.28)** |  | 1.54 (0.92, 2.58) |
| Model 1^†^ |  | **2.11 (1.51, 2.95)** |  | **2.22 (1.47, 3.37)** |  | **1.93 (1.11, 3.36)** |
| Model 2^‡^ |  | **2.13 (1.52, 2.98)** |  | **2.26 (1.49, 3.42)** |  | **1.91 (1.09, 3.34)** |
| **Smoker P1^2^** | 735 |  | 626 |  | 109 |  |
| Unadjusted |  | **1.45 (1.10, 1.91)** |  | **1.75 (1.24, 2.46)** |  | 0.88 (0.54, 1.44) |
| Model 1^†^ |  | **1.50 (1.10, 2.03)** |  | **1.75 (1.22, 2.53)** |  | 1.05 (0.62, 1.78) |
| Model 2^‡^ |  | **1.53 (1.13, 2.07)** |  | **1.80 (1.25, 2.60)** |  | 1.05 (0.62, 1.79) |
| **Other smoker^3^** | 1346 |  | 1230 |  | 116 |  |
| Unadjusted |  | 0.89 (0.69, 1.15) |  | 1.09 (0.80, 1.48) |  | 0.82 (0.50, 1.33) |
| Model 1^†^ |  | 1.11 (0.84, 1.45) |  | 1.17 (0.84, 1.62) |  | 0.98 (0.59, 1.64) |
| Model 2^‡^ |  | 1.12 (0.85, 1.47) |  | 1.17 (0.85, 1.62) |  | 1.01 (0.61, 1.69) |
| **Ex-smoker** | 4065 |  | 3681 |  | 384 |  |
| Unadjusted |  | **0.70 (0.59, 0.84)** |  | 0.81 (0.65, 1.01) |  | **0.65 (0.47, 0.88)** |
| Model 1^†^ |  | 0.89 (0.73, 1.07) |  | 0.93 (0.74, 1.17) |  | 0.82 (0.59, 1.15) |
| Model 2^‡^ |  | 0.90 (0.74, 1.08) |  | 0.93 (0.74, 1.17) |  | 0.83 (0.60, 1.17) |
| **Never smoker** | 8354 | Reference | 7353 | Reference | 1001 | Reference |
| 1. A smoker at the first ANA for P2 who was not smoking at the first ANA for P1   2. A smoker at the first ANA for P1 who stopped before the first ANA for P2  3. A smoker later in P1 or between pregnancies; not smoking at the first ANA for P1 or P2  **† Model 1 (adjusts for confounders):** Adjusted for maternal age, BMI, educational attainment, employment status, partnership status, folate supplementation and infertility treatment at the start of the first pregnancy, gestational diabetes mellitus and gestational hypertension recorded during the first pregnancy, SGA birth in the first pregnancy (not in the stratified analysis), maternal ethnicity and the length of the interpregnancy interval **‡ Model 2 (adjusts for confounders and mediators):** Adjusted for maternal age, BMI, educational attainment, employment status, partnership status, folate supplementation and infertility treatment at the start of the first pregnancy, gestational diabetes mellitus and gestational hypertension recorded during the second pregnancy, SGA birth in the first pregnancy (not in the stratified analysis), maternal BMI at the start of the second pregnancy, maternal ethnicity and the length of the interpregnancy interval **Abbreviations:** ANA, antenatal appointment; BMI, body mass index; CI, confidence interval; P1, first pregnancy; P2 second pregnancy; SGA, small for gestational age (<10^th^ percentile) | | | | | | |

**“Model 1 adjusts for confounders and in the full sample shows the odds of SGA birth in the second pregnancy adjusting for maternal age, BMI, educational attainment, employment status, partnership status, folate supplementation and infertility treatment at the start of the first pregnancy, gestational diabetes mellitus and gestational hypertension recorded during the first pregnancy, SGA birth in the first pregnancy, maternal ethnicity and the length of the interpregnancy interval.**

**Compared to never smokers, there are increased odds of SGA birth in the second pregnancy for heavier smokers ((adjusted odds ratio (aOR) 3.54 [95% confidence interval (CI) 2.55, 4.92]), smokers (2.44 [1.89, 3.15]), smoker increased (2.70 [1.92, 3.82]), smoker reduced (2.44 [1.68, 3.54]), smokers P2 (2.11 [1.51, 2.95]) and smokers P1 (stopped before the first ANA P2) (1.50 [1.10, 2.03]). Other smokers, (smokers later in P1 or between pregnancies but not smoking at the first ANA of P1 or P2) or ex-smokers did not have increased odds of SGA birth in the second pregnancy compared to never smokers ((1.11 [0.84, 1.45]) and (0.89 [0.73, 1.07]) respectively).**

**Model 1 in the sample which excludes women whose first child was born SGA makes the same adjustments described above, with the exception of an adjustment for previous SGA birth. Compared to never smokers, there were increased odds of new SGA for heavier smokers (3.53 [2.32, 5.38]), smokers (2.43 [1.75, 3.39]), smoker increased (2.84 [1.82, 4.44]), smoker reduced (2.98 [1.90, 4.67]), smokers P2 (2.22 [1.47, 3.37]) and smokers P1 (stopped before the first ANA P2) (1.75 [1.22, 2.53]). Other smokers (smokers later in P1 or between pregnancies but not smoking at the first ANA of P1 or P2) or ex-smokers did not have increased odds of new SGA compared to never smokers ((1.17 [0.84, 1.62]) and (0.93 [0.74, 1.17]) respectively).**

**Model 1 in the sample where there was SGA birth in the first pregnancy, shows the odds of recurrent SGA birth. The same adjustments were made as described above.**

**Compared to never smokers, there were increased odds of recurrent SGA birth in the second pregnancy heavier smokers (3.34 [1.96, 5.68]), smokers (2.34 [1.56, 3.51]), smoker increased (2.42 [1.41, 4.16]) and smokers P2 (1.93 [1.11, 3.36]). Compared to never smokers, there was no increase in the odds of recurrent SGA birth for smoker reduced (1.75 [0.94, 3.25]), smokers P1 (stopped before the first ANA P2) (1.05 [0.62, 1.78]), other smokers (smokers later in P1 or between pregnancies but not smoking at the first ANA of P1 or P2) (0.98 [0.59, 1.64]) or ex-smokers (0.82 [0.59, 1.15]).**

**Model 2 adjusts for confounders and mediators and in the full sample shows the odds of SGA birth in the second pregnancy adjusting for maternal age, BMI, educational attainment, employment status, partnership status, folate supplementation and infertility treatment at the start of the first pregnancy, gestational diabetes mellitus and gestational hypertension recorded during the second pregnancy, maternal BMI at the start of the second pregnancy, SGA birth in the first pregnancy, maternal ethnicity and the length of the interpregnancy interval.**

**Compared to never smokers, there were increased odds of SGA birth in the second pregnancy for heavier smokers (3.57 [2.57, 4.97]), smokers (2.43 [1.88, 3.14]), smoker increased (2.75 [1.94, 3.88]), smoker reduced (2.50 [1.72, 3.63]), smokers P2 (2.13 [1.52, 2.98]) and smokers P1 (stopped before the first ANA P2) (1.53 [1.13, 2.07]). Other smokers, (smokers later in P1 or between pregnancies but not smoking at the first ANA of P1 or P2) or ex-smokers did not have increased odds of SGA birth in the second pregnancy compared to never smokers ((1.12 [0.85, 1.47]) and (0.90 [0.74, 1.08]) respectively).**

**Model 2 in the sample excluding women whose first child was born SGA makes the same adjustments described above, with the exception of an adjustment for previous SGA birth. Compared to never smokers, there were increased odds of new SGA for heavier smokers (3.52 [2.31, 5.37]), smokers (2.47 [1.77, 3.44]), smoker increased (2.87 [1.84, 4.49]), smoker reduced (3.05 [1.95, 4.78]), smokers P2 (2.26 [1.49, 3.42]) and smokers P1 (stopped before the first ANA P2) (1.80 [1.25, 2.60]). Other smokers (smokers later in P1 or between pregnancies but not smoking at the first ANA of P1 or P2) or ex-smokers did not have increased odds of new SGA compared to never smokers ((1.17 [0.85, 1.62]) and (0.93 [0.74, 1.17]) respectively).**

**Model 2 in the sample with SGA birth in the first pregnancy shows the odds of recurrent SGA birth. The same adjustments were made as described above.**

**Compared to never smokers, there were increased odds of recurrent SGA birth in the second pregnancy heavier smokers (3.54 [2.07, 6.08]), smokers (2.23 [1.48, 3.35]), smoker increased (2.51 [1.45, 4.32]) and smokers P2 (1.91 [1.09, 3.34]). Compared to never smokers, there was no increase in the odds of recurrent SGA birth for smoker reduced (1.82 [0.97, 3.39]), smokers P1 (stopped before the first ANA P2) (1.05 [0.62, 1.79]), other smokers (smokers later in P1 or between pregnancies but not smoking at the first ANA of P1 or P2) (1.01 [0.61, 1.69]) or ex-smokers (0.83 [0.60, 1.17]).**

**The full results for Model 1 in the full sample (Table 4) are given in Table 5.”**

**Supplementary Table 1, showing the sensitivity analysis undertaken using different minimal adjustment sets, is reproduced below and the following paragraph has been added to the Results section of the Manuscript [lines: 336-340]:**

**“Sensitivity analysis for Model 1 was run using the other minimal adjustment sets identified by DAGitty, as described in the Methods section above. The results of this analysis (Supplementary Table 1) show only minor differences in the adjusted odds ratios for Model 1 whichever minimal adjustment set is used, noting slight differences in the numbers of missing observations across the different models.”**

**Supplementary Table 1: Sensitivity analysis showing the effect of using different minimal adjustment sets on the adjusted odds ratios calculated in Model 1 in the full sample**

| **Model^¶^** | **Heavier smoker** | **Smoker** | **Smoker increased** | **Smoker reduced** | **Smoker P2^1^** | **Smoker P1^2^** | **Other smoker^3^** | **Ex-smoker** |
| --- | --- | --- | --- | --- | --- | --- | --- | --- |
| Adjusted odds ratios (aORs) | | | | | | | | |
| 1^†^ | 3.55 | 2.44 | 2.70 | 2.44 | 2.11 | 1.50 | 1.11 | 0.89 |
| Differences to the aORs for Model 1 in each of the models run with different adjustment sets^¶^ | | | | | | | | |
| A^†^ | 0.04 | -0.03 | 0.02 | 0.06 | 0.01 | 0.03 | 0.01 | 0.00 |
| B^†^ | -0.01 | -0.03 | 0.01 | 0.04 | 0.01 | 0.03 | 0.01 | 0.00 |
| C^†^ | 0.00 | -0.03 | 0.03 | 0.05 | 0.01 | 0.03 | 0.01 | 0.01 |
| D^‡^ | 0.13 | 0.04 | 0.19 | 0.06 | 0.07 | 0.00 | -0.01 | 0.00 |
| E^‡^ | 0.02 | -0.01 | 0.11 | 0.05 | 0.05 | -0.01 | -0.01 | 0.00 |
| F^‡^ | 0.15 | 0.05 | 0.21 | 0.07 | 0.08 | 0.00 | 0.00 | 0.00 |
| G^‡^ | 0.14 | 0.04 | 0.20 | 0.07 | 0.08 | 0.00 | 0.00 | 0.00 |
| H^‡^ | 0.04 | 0.00 | 0.14 | 0.06 | 0.05 | 0.00 | -0.01 | 0.00 |
| I^‡^ | 0.04 | 0.00 | 0.13 | 0.06 | 0.05 | -0.01 | -0.01 | 0.00 |
| J^§^ | 0.16 | 0.03 | 0.22 | 0.11 | 0.10 | 0.03 | 0.01 | 0.01 |
| K | 0.05 | -0.01 | 0.15 | 0.09 | 0.07 | 0.02 | 0.01 | 0.01 |
| L^§^ | 0.18 | 0.04 | 0.25 | 0.12 | 0.10 | 0.03 | 0.01 | 0.01 |
| M^§^ | 0.17 | 0.03 | 0.24 | 0.12 | 0.10 | 0.03 | 0.01 | 0.01 |
| N | 0.08 | 0.00 | 0.18 | 0.11 | 0.08 | 0.02 | 0.01 | 0.01 |
| O | 0.07 | -0.01 | 0.18 | 0.11 | 0.08 | 0.02 | 0.01 | 0.01 |
| P^§^ | 0.13 | 0.03 | 0.23 | 0.10 | 0.09 | 0.03 | 0.01 | 0.01 |
| Q | 0.02 | -0.01 | 0.16 | 0.08 | 0.07 | 0.02 | 0.00 | 0.01 |
| R^§^ | 0.15 | 0.04 | 0.25 | 0.11 | 0.10 | 0.03 | 0.01 | 0.01 |
| S^§^ | 0.14 | 0.03 | 0.25 | 0.11 | 0.10 | 0.03 | 0.01 | 0.01 |
| T | 0.04 | 0.00 | 0.19 | 0.11 | 0.07 | 0.02 | 0.01 | 0.01 |
| U | 0.04 | -0.01 | 0.18 | 0.10 | 0.07 | 0.02 | 0.01 | 0.01 |
| 1. A smoker at the first ANA for P2 who was not smoking at the first ANA for P1  2. A smoker at the first ANA for P1 who stopped before the first ANA for P2 3. A smoker later in P1 or between pregnancies; not smoking at the first ANA for P1 or P2  † These models had missing data for variables in 76 records  ‡ These models had missing data for variables in 5 records  § These models had missing data for variables in 1 record  ¶ All models adjusted for maternal ethnicity, length of the IPI, highest level of maternal educational attainment and previous SGA birth. The models additionally adjusted for:  **Model 1** Maternal employment, P1 BMI, P1 GDM, P1 age, P1 folic acid supplementation, P1 gestational hypertension, P1 infertility treatment, P1 partnership status  **Model A** Maternal employment, P1 BMI, P1 GDM, P1 age, P1 folic acid supplementation, P1 gestational hypertension, P1 partnership status, P2 BMI, P2 infertility treatment, P2 partnership status  **Model B** Maternal employment, P1 BMI, P1 age, P1 folic acid supplementation, P1 gestational hypertension, P1 infertility treatment, P1 partnership status, P2 BMI, P2 GDM  **Model C** Maternal employment, P1 BMI, P1 age, P1 folic acid supplementation, P1 gestational hypertension, P1 partnership status, P2 BMI, P2 GDM, P2 infertility treatment, P2 partnership status  **Model D** P1 BMI, P1 GDM, P1 age, P1 folic acid supplementation, P1 gestational hypertension, P1 infertility treatment, P1 partnership status, P2 age  **Model E** P1 BMI, P1 GDM, P1 age, P1 gestational hypertension, P1 infertility treatment, P1 partnership status, P2 age, P2 folic acid supplementation  **Model F** P1 BMI, P1 GDM, P1 folic acid supplementation, P1 gestational hypertension, P1 infertility treatment, P1 partnership status, P2 age, P2 partnership status  **Model G** P1 BMI, P1 GDM, P1 folic acid supplementation, P1 gestational hypertension, P1 infertility treatment, P2 age, P2 infertility treatment, P2 partnership status  **Model H** P1 BMI, P1 GDM, P1 gestational hypertension, P1 infertility treatment, P1 partnership status, P2 age, P2 folic acid supplementation, P2 partnership status  **Model I** P1 BMI, P1 GDM, P1 gestational hypertension, P1 infertility treatment, P2 age, P2 folic acid supplementation, P2 infertility treatment, P2 partnership status  **Model J** P1 GDM, P1 age, P1 folic acid supplementation, P1 gestational hypertension, P1 infertility treatment, P1 partnership status, P2 BMI, P2 age  **Model K** P1 GDM, P1 age, P1 gestational hypertension, P1 infertility treatment, P1 partnership status, P2 BMI, P2 age, P2 folic acid supplementation  **Model L** P1 GDM, P1 folic acid supplementation, P1 gestational hypertension, P1 infertility treatment, P1 partnership status, P2 BMI, P2 age, P2 partnership status  **Model M** P1 GDM, P1 folic acid supplementation, P1 gestational hypertension, P2 BMI, P2 age, P2 infertility treatment, P2 partnership status  **Model N** P1 GDM, P1 gestational hypertension, P1 infertility treatment, P1 partnership status, P2 BMI, P2 age, P2 folic acid supplementation, P2 partnership status  **Model O** P1 GDM, P1 gestational hypertension, P2 BMI, P2 age, P2 folic acid supplementation, P2 infertility treatment, P2 partnership status  **Model P** P1 age, P1 folic acid supplementation, P1 gestational hypertension, P1 infertility treatment, P1 partnership status, P2 BMI, P2 GDM, P2 age  **Model Q** P1 age, P1 gestational hypertension, P1 infertility treatment, P1 partnership status, P2 BMI, P2 GDM, P2 age, P2 folic acid supplementation  **Model R** 1 folic acid supplementation, P1 gestational hypertension, P1 infertility treatment, P1 partnership status, P2 BMI, P2 GDM, P2 age, P2 partnership status  **Model S** P1 folic acid supplementation, P1 gestational hypertension, P2 BMI, P2 GDM, P2 age, P2 infertility treatment, P2 partnership status  **Model T** P1 infertility treatment, P1 partnership status, P2 BMI, P2 GDM, P2 age, P2 folic acid supplementation, P2 partnership status  **Model U** P1 gestational hypertension, P2 BMI, P2 GDM, P2 age, P2 folic acid supplementation, P2 infertility treatment, P2 partnership status  **Abbreviations:** ANA, antenatal appointment; BMI, body mass index; P1, first pregnancy; P2, second pregnancy; SGA, small for gestational age (< 10^th^ percentile); OR, odds ratio; aOR, adjusted odds ratio; CI, confidence interval. | | | | | | | | |

Table 3:

Could the maternal characteristics at the start of pregnancy 2 be expressed as univariate odds ratios as per Table 6?

This would usually allow the reader to examine whether there is a statistical basis for adjustment on the covariates present at the beginning of the second pregnancy. Notwithstanding that there may be other reasons for inclusion of a covariate based on content knowledge or prior research.

Why did the authors present this table allowing the reader to examine the relationship between maternal characteristics at the start of the second pregnancy when these are not the covariates that they use to calculate the adjusted odds ratios?

**Thank you, this has now been done and included as a new Table 3, reproduced below. We agree that this is a really useful way of presenting the information and believe it clearly supports the selection of the covariates via our DAG into our models.**

**Table 3: Univariate odds of small for gestational age birth (< 10^th^ percentile) in the second pregnancy in the full sample, by maternal characteristics recorded at the start of or during the first and second pregnancies**

| **Maternal Characteristics** | **First pregnancy** | | **Second pregnancy** | |
| --- | --- | --- | --- | --- |
|  | **n** | **OR (95% CI)** | **n** | **OR (95% CI)** |
| **Age category  (ref = 25-34 years)** |  |  |  |  |
| < 18 years | 1005 | **2.2 (1.77, 2.73)** | 63 | 1.97 (0.89, 4.33) |
| 18-24 years | 5793 | **1.66 (1.45, 1.89)** | 3913 | **1.65 (1.44, 1.89)** |
| 35-39 years | 845 | 0.99 (0.73, 1.36) | 2386 | 1.01 (0.84, 1.22) |
| 40 years and over | 47 | 1.65 (0.59, 4.60) | 326 | 0.81 (0.49, 1.35) |
| **BMI category  (ref = normal weight (18.5-24.9 kg/m^2^))** |  |  |  |  |
| Underweight (<18.5 kg/m^2^) | 628 | **1.93 (1.51, 2.46)** | 473 | **2.30 (1.77, 2.98)** |
| Overweight (25-29.9 kg/m^2^) | 4106 | **0.78 (0.67, 0.91)** | 4516 | **0.76 (0.65, 0.88)** |
| Obese (≥30 kg/m^2^) | 2241 | **0.71 (0.58, 0.87)** | 3282 | **0.69 (0.58, 0.82)** |
| **Highest level of education  (ref = degree level)** |  |  |  |  |
| College level | 6272 | **1.20 (1.02, 1.41)** | 6923 | **1.31 (1.12, 1.52)** |
| Secondary or below | 5531 | **1.84 (1.57, 2.15)** | 4272 | **1.95 (1.66, 2.28)** |
| **Folic acid status  (ref = taking prior to pregnancy)** |  |  |  |  |
| Started once pregnancy confirmed | 9624 | **1.55 (1.35, 1.79)** | 9986 | **1.47 (1.27, 1.70)** |
| Not taking folic acid | 1454 | **2.17 (1.76, 2.68)** | 1489 | **2.43 (1.99, 2.98)** |
| Not in employment | 3458 | **2.11 (1.85, 2.40)** | 5530 | **1.91 (1.69, 2.15)** |
| **Received infertility treatment** | 680 | 0.87 (0.63, 1.21) | 514 | 0.75 (0.51, 1.11) |
| **Lone parent** | 1450 | **1.42 (1.17, 1.72)** | 1057 | **1.44 (1.16, 1.78)** |
| **Gestational diabetes mellitus** | 292 | **0.43 (0.22, 0.84)** | 425 | 0.82 (0.54, 1.24) |
| **Gestational hypertension** | 425 | 0.78 (0.51, 1.19) | 188 | **1.74 (1.10, 2.74)** |
|  | **Non pregnancy specific** | | | |
|  | **n** | **OR (95% CI)** | | |
| **Maternal Ethnicity  (ref = White)** |  |  | | |
| Mixed | 196 | 0.98 (0.55, 1.77) | | |
| Asian | 987 | **2.61 (2.16, 3.15)** | | |
| Black/African/Caribbean | 247 | 1.47 (0.94, 2.29) | | |
| Chinese | 99 | 1.14 (0.53, 2.48) | | |
| Other | 173 | **1.75 (1.07, 2.86)** | | |
| Not known | 551 | 0.93 (0.65, 1.33) | | |
| **Length of the IPI  (ref = 12 to < 24 months)** |  |  | | |
| < 12 months | 2937 | **1.32 (1.10, 1.57)** | | |
| 24 to < 36 months | 3842 | **1.19 (1.01, 1.41)** | | |
| 36 months or more | 4117 | **1.39 (1.19, 1.63)** | | |
| **Previous SGA birth** | 2067 | **6.67 (5.86, 7.58)** | | |
| **Abbreviations:** BMI, body mass index; IPI, interpregnancy interval (from the birth of the first infant to the conception of the second); SGA, small for gestational age (< 10^th^ percentile); OR, odds ratio; CI, confidence interval. | | | | |

Table 5:

In my opinion this sensitivity analysis adds nothing to the study. I would omit it or move it to a supplementary analysis.

**Thank you, this Table has now been removed, along with references to it within the manuscript.**

Discussion:

As previously discussed, I do not think that there is enough a priori evidence for the authors main hypothesis. Therefore, I feel their conclusion at the beginning of the discussion section is too strong.

**Here we have changed the wording to read “smoking at the start of the first pregnancy may be an important factor in shaping the risk of SGA birth in the second pregnancy” [lines 349-350].**

This paper is a good exploration of their data set but given the nature of the smoking status variable a sensitivity analysis for all variations of the smoking status that includes any smoking between pregnancies would lead to a null finding.

**Thank you, the Table with the sensitivity analysis has now been removed as noted above.**

The authors own discussion acknowledges the issues with patient recall of smoking around pregnancy with discordance rates between 19-39%. Further, it seems that women with higher risk factors for SGA were more likely to have discordance between reported and actual rates of smoking in pregnancy.

**Thank you, whilst we acknowledge the reviewer’s point and agree that there may be some discordance here, we hope that raising it in the Discussion has drawn attention to this and that mentioning it in the strengths and limitations section will have further flagged this concern. The study referenced in our discussion did find that retrospective recall of smoking in pregnancy to be stable; in our study women are asked about smoking status at their antenatal appointments and the response recorded at that stage by a midwife. We have added the following sentence to this part of the discussion [lines 378-380] :**

**“In our study women were asked for their smoking status at the start of each pregnancy and the responses recorded at that time, which means that recall bias is unlikely.”**

Conclusion:

I would recommend a major revision and further explanation of the multivariate models before publication. I would be happy to review a revised draft of this paper.

Reviewer #2: This is a well conducted and described study. I have a few minor comments:
- the counts of individuals in each of the exposure categories - included in the description of the exposure variable in the first column of Table 1 - are results and should not be included in the methods section. They are already included in the first row of table 2 in the results section, so it would be sufficient to just remove them from table 1.
**Thank you, the numbers have been removed from Table 1.**

- please remove the ± designation wherever you report SDs and instead report the means followed by the SDs in brackets.
**Thank you we have removed the ± designation and now reported means followed by SDs in brackets.**

- in Table 2 where you report proportions (e.g. of BMI, ethnicity, education categories), it is unclear what the numbers included in brackets represent. Additionally for the last row where you report n's again, it would be better to instead report the numbers of missing, since the n's are already included in the column headers.
**Thank you we have now updated the table as suggested.**

- because the results in Table 2 are purely descriptive (not inferential), you should not be reporting any confidence intervals here. You should simply report mean (SD) or median (IQR) for continuous variables and n (%) for categorical ones (where n is the count of each category and % is the percentage of that count out of the number of individuals in the main exposure/column)
**Thank you, this has now been amended. We have also added a column to show *p-*values and included the following paragraph to the statistical analysis section of the manuscript, and an appropriate footnote in Table 2 [lines 160-161] :**

**“Unadjusted comparisons were carried out using Chi-squared tests for categorical variables and ANOVA for continuous variables”**

- relating to the comment above, please distinguish clearly between N, the denominator for a proportion, and n the numerator; so for example, you shouldn't have two columns both labelled n as you do in table 3. The first n is the denominator and should be labelled capital N.
**Thank you, Table 3 has now been replaced with a different table based on a suggestion made by the other reviewer.**
